# Optimal stapler cartridge selection according to the thickness of the pancreas in distal pancreatectomy

**DOI:** 10.1097/MD.0000000000004441

**Published:** 2016-09-02

**Authors:** Hongbeom Kim, Jin-Young Jang, Donghee Son, Seungyeoun Lee, Youngmin Han, Yong Chan Shin, Jae Ri Kim, Wooil Kwon, Sun-Whe Kim

**Affiliations:** a Department of Surgery and Cancer Research Institute, College of Medicine, Seoul National University; b Department of Mathematics and Statistics, Sejong University, Seoul, Korea.

**Keywords:** compression, distal pancreatectomy, fistula, pancreas, risk factor, surgical stapler, thickness

## Abstract

Stapling is a popular method for stump closure in distal pancreatectomy (DP). However, research on which cartridges are suitable for different pancreatic thickness is lacking. To identify the optimal stapler cartridge choice in DP according to pancreatic thickness.

From November 2011 to April 2015, data were prospectively collected from 217 consecutive patients who underwent DP with 3-layer endoscopic staple closure in Seoul National University Hospital, Korea. Postoperative pancreatic fistula (POPF) was graded according to International Study Group on Pancreatic Fistula definitions. Staplers were grouped based on closed length (CL) (Group I: CL ≤ 1.5 mm, II: 1.5 mm < CL < 2 mm, III: CL ≥ 2 mm). Compression ratio (CR) was defined as pancreas thickness/CL. Distribution of pancreatic thickness was used to find the cut-off point of thickness which predicts POPF according to stapler groups.

POPF developed in 130 (59.9%) patients (Grade A; n = 86 [66.1%], B; n = 44 [33.8%]). The numbers in each stapler group were 46, 101, and 70, respectively. Mean thickness was higher in POPF cases (15.2 mm vs 13.5 mm, *P* = 0.002). High body mass index (*P* = 0.003), thick pancreas (*P* = 0.011), and high CR (*P* = 0.024) were independent risk factors for POPF in multivariate analysis. Pancreatic thickness was grouped into <12 mm, 12 to 17 mm, and >17 mm. With pancreatic thickness <12 mm, the POPF rate was lowest with Group II (I: 50%, II: 27.6%, III: 69.2%, *P* = 0.035).

The optimal stapler cartridges with pancreatic thickness <12 mm were those in Group II (Gold, CL: 1.8 mm). There was no suitable cartridge for thicker pancreases. Further studies are necessary to reduce POPF in thick pancreases.

## 1 Introduction

Stapling is a popular method for stump closure in distal pancreatectomy (DP). In some studies, staplers had similar outcome to hand-sewn closure.^[1]^ Another review concluded that stapling closure had more favorable results than hand-sewn closure.^[2]^ Although these are encouraging results, postoperative pancreatic fistula (POPF) still occurs at 24% to 39%.^[2–5]^ In previous studies, the suggested risk factors of POPF after DP were body mass index (BMI),^[6]^ pancreatic thickness,^[7]^ and pancreatic texture.^[8]^ Among these risk factors, thickness is an adjustable factor influenced by the stapler. However, research is lacking on the selection of cartridges in DP according to pancreatic thickness.

The purpose of this study was to investigate risk factors for POPF following DP, and to identify the optimal stapler cartridge to use according to the thickness of the pancreas.

## 2 Materials and methods

### 2.1 Patient characteristics and definition of pancreatic fistula

From November 2011 to April 2015, 217 consecutive patients underwent open or laparoscopic DP with 3-layer endoscopic stapler closure at Seoul National University Hospital, Korea. Patient characteristics were reviewed for age, sex, BMI, pathologic diagnosis, and operation method. Clinicopathological data and radiological images were prospectively collected in electronic medical record form. Serum and drain amylase were checked at 3 days after the operation. The texture of the pancreatic parenchyma was classified into 2 groups, soft and hard by the surgeon during the operation. The diagnosis and grade of postoperative pancreatic fistulae were recorded prospectively by the research coordinator, who did not join the operation according to the criteria established by the International Study Group on Pancreatic Fistula.^[9]^ The study was approved by the institutional review board of Seoul National University hospital (IRB No. 1506-105-682).

### 2.2 Operation and stapler

In laparoscopic DP cases, the patient was laid in the supine position under general anesthesia. After pneumoperitoneum was established, a 12 mm trocar for the videoscope was inserted into the peritoneal cavity through a subumbilical incision. The other trocars were then inserted. The operation was performed through 4 ports. The greater omentum was separated from the transverse colon and the pancreas was exposed. The upper border of the pancreas was dissected and the splenic artery was identified. The splenic artery was ligated with Hem-o-lock clips (Teleflex, Morrisville, NC) and divided. The splenocolic and splenorenal ligaments were divided. The pancreas was resected with an endoscopic 3-layer stapler. Before firing, the pancreas was compressed for 20 to 30 s with the stapler. The resected specimen was extracted through a small incision created by extending the subumbilical port incision. After meticulous hemostasis and warm saline irrigation, a Jackson-Pratt (JP) drain was placed in the pancreatic stump. The surgical wound was closed in layers. An aseptic dressing was applied on it. In open surgery cases, upper mid line incision was made. Operative procedures were identical to those of laparoscopic DP.

Two products of endoscopic stapler bodies, Echelon Flex (Ethicon Endosurgery, Cincinnati, OH) and Endo GIA (Covidien Medtronic, Plymouth, MN) were used. The values of closed length (CL) were referred in the product documentation. Ethicon cartridges were supplied in the colors, white, gold, green, and black with CL 1.0, 1.8, 2.0, and 2.3 mm, respectively. In CL range of Covidien cartridges, tan, purple, and black cartridges were 0.88 to 1.8, 1.5 to 2.25, and 2.25 to 3.0 mm, respectively. To compare stapler effect regardless of its manufacturer, cartridges were grouped into 3 groups according to the CL: below 1.5 mm, between 1.5 and 2 mm, and above 2 mm.

### 2.3 Measurement of pancreas thickness and compression ratio

**Figure 1:**
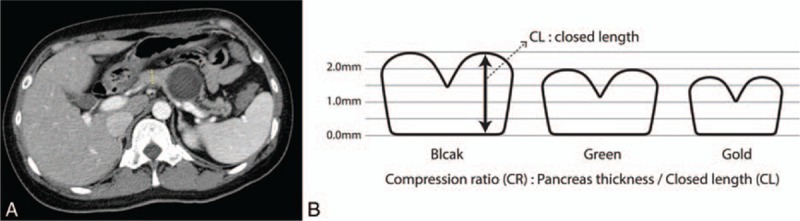
(A) Thickness measurement at resection line: Pancreatic thickness was measured at the resection line in preoperative computed tomography by 1 of the researchers. (B) Compression ratio: CR was defined as pancreas thickness divided by CL for comparing degree of compression. CL = closed length.

**Figure 2:**
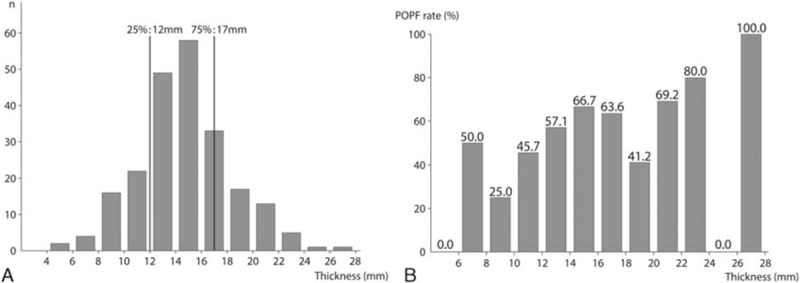
(A) Distribution and subgrouping of thickness: The 25th percentile of thickness was 12 mm and the 75th percentile was 17 mm. Thickness was divided into 3 subgroups, below 12 mm, between 12 and 17 mm, and above 17 mm. (B) POPF rate according to the thickness: POPF rate increased as thickness increased. POPF = postoperative pancreatic fistula.

Pancreatic thickness was measured at the resection line in preoperative computed tomography (CT) by 1 researcher who did not know the POPF result (Fig. 1). Resection line was evaluated with postoperative CT at 4 days after the operation. To compare the degree of compression, the compression ratio (CR) was defined as the pancreas thickness divided by CL. CR was the thickness ratio between pre and postcompression (Fig. 1). To figure out the optimal stapler according to the thickness, thickness was divided 3 subgroups: below 25th percentile of distribution of thickness, between the 25th percentile and the 75th percentile, and above the 75th percentile (Fig. 2).

### 2.4 Statistical analysis

All statistical analyses were performed with SPSS version 21.0 (SPSS Inc., Chicago, IL). Nominal data were compared with the chi-squared test and continuous data with Student *t* test. Binominal logistic regression analysis was used to find risk factors for POPF and the odds ratio (OR) of the optimal stapler. Variables which were statistically significant in the univariate analysis were used in the multivariate analysis. *P* values of <0.05 were considered statistically significant.

## 3 Results

### 3.1 Demographics

The characteristics of the patients are listed in Table 1. The 217 patients included 92 men (42.3%) and 125 women (57.6%), of mean age 60.3 ± 13.5 years. Benign disease was present in 130 cases (59.9%) and malignant disease in 87 cases (40.1%). The most common pathologic diagnosis was pancreatic ductal adenocarcinoma. A soft pancreas was seen in 168 (77.4%) of cases. Mean thickness of the pancreas was 14.6 ± 3.9 mm, and the median value was 15 mm. Figure 1 shows the distribution of thickness. The 25th percentile of thickness was 12 mm and the 75th percentile was 17 mm. Six types of stapler cartridges were used. In Group II staplers, between 1.5 and 2.0 mm of CL, the gold was most commonly used (Table 2).

**Table 1:**
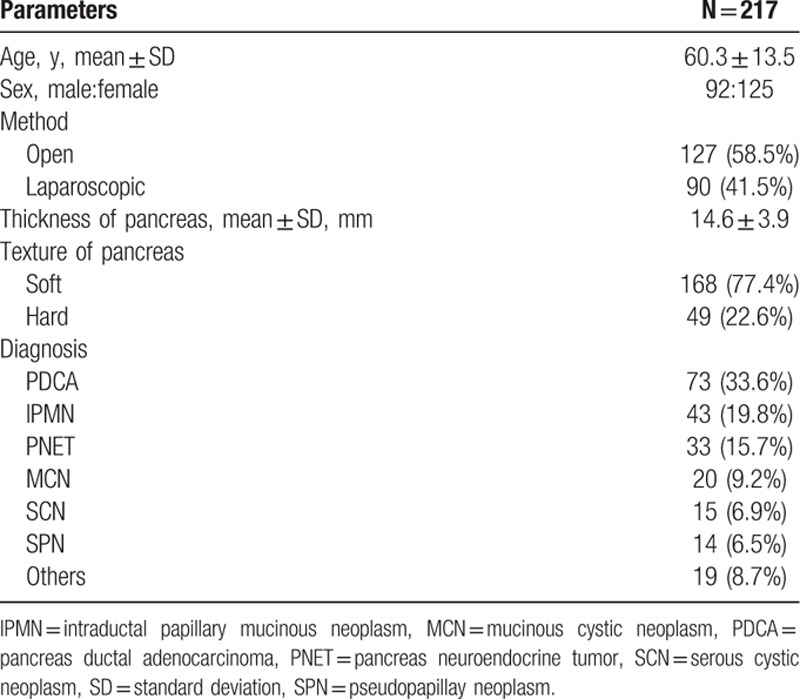
Patients’ characteristics.

**Table 2:**
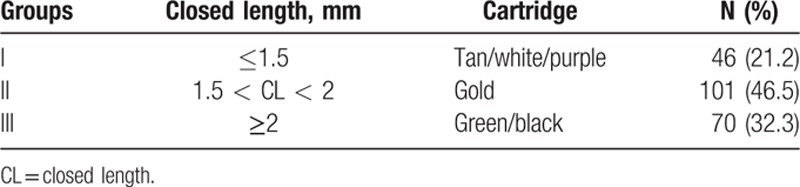
Stapler cartridges.

### 3.2 Incidence and risk factors of POPF

Among the 217 patients, POPF developed in 130 patients (59.9%). Grades A, B, and C were 86 (39.6%), 44 (20.2%), and 0 (0%), respectively. The rate of clinically relevant POPF was 20.2%. In the POPF group, BMI was higher than in the POPF (−) group (24.1 vs 22.7, *P* = 0.001). Hard pancreases had a tendency toward development of POPF compared with soft pancreases, but this was not statistically insignificant (69.4% vs 57.1%, *P* = 0.124). Stapler group and mean of CL did not show any difference between POPF (+) and POPF (−) groups. In multivariate analysis, the risk factors for POPF were high BMI, thick pancreas, and high CR (Table 3).

**Table 3:**
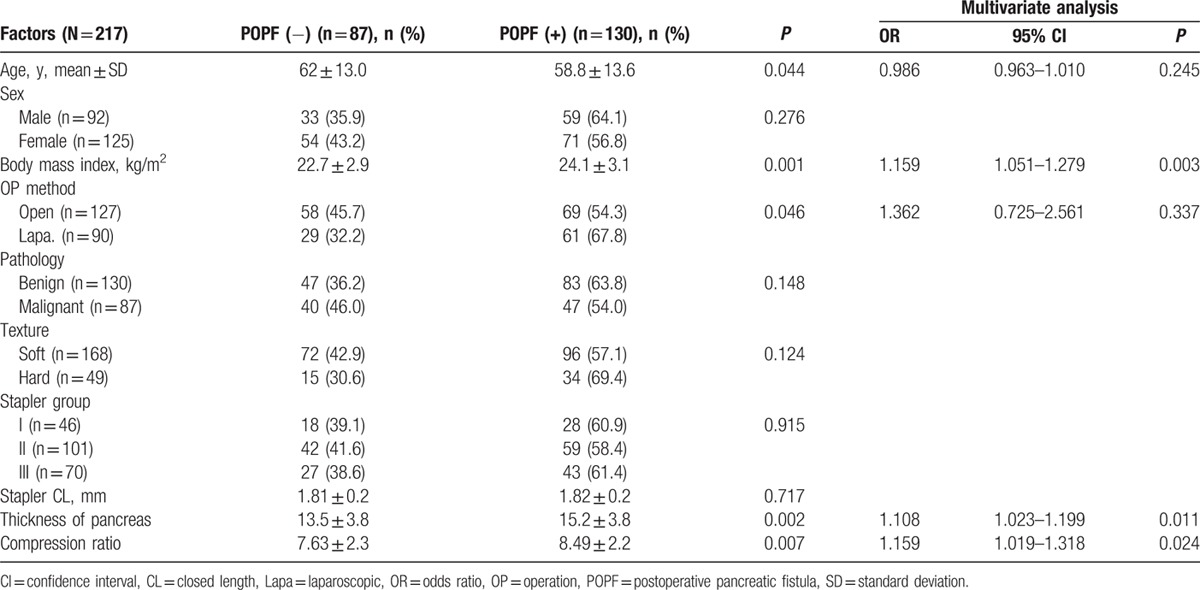
Risk factors for POPF.

### 3.3 POPF according to the thickness

Figure 2 shows the POPF rate according to the thickness. The POPF rate increased as thickness increased. The median value of thickness, 15 mm was regarded as the division point between thick and thin pancreas. Table 4 shows different risk factors according to the thickness. In thickness < 15 mm, high BMI was a significant risk factors in multivariate analysis (*P* = 0.013), thickness was a risk factor in univariate analysis (*P* = 0.043) but was only marginally significant in multivariate analysis (*P* = 0.061). In thick pancreases above 15 mm, the only risk factor was high BMI.

**Table 4:**
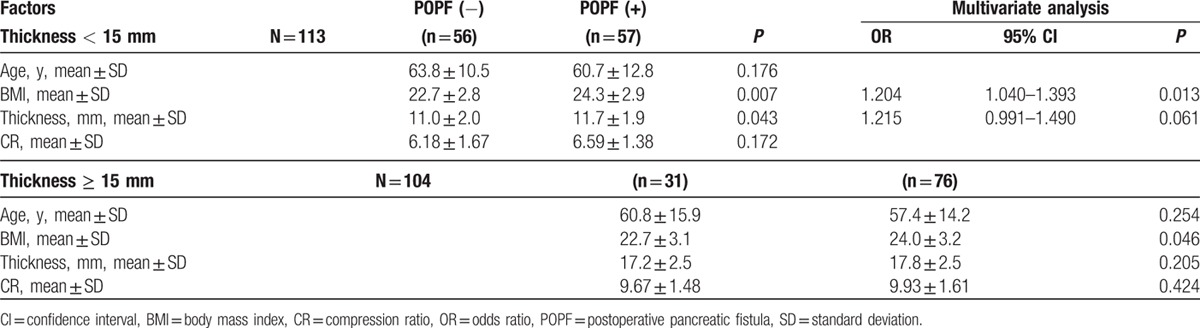
Risk factors according to the thickness.

### 3.4 Optimal stapler and CR according to the thickness

**Figure 3:**
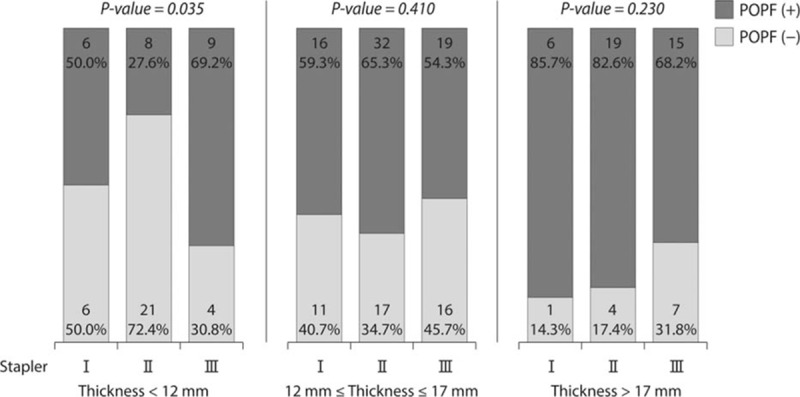
POPF rate according to the stapler group in subgroup analysis: In the thickness <12 mm group, when using Group II (gold) staplers, POPF rate was lowest. In the thickness >17 mm group, the POPF rate tended to be lower when a longer stapler was used, but this was not statistically significant. POPF = postoperative pancreatic fistula.

Patients were split into 3 subgroups by pancreatic thickness: below 12 mm, between 12 and 17 mm, and above 17 mm (Fig. 1). In the <12 mm group, when using Group II (Gold) staplers, the POPF rate was lowest (50.0% vs 27.6% vs 69.2%, *P* = 0.035) (Fig. 3). The OR between Group II and the other groups was 3.937 (95% of OR: 1.257–12.332, *P* = 0.019). However, the mean of CR did not show any difference (POPF: 5.51, no POPF: 5.50, *P* = 0.951). In the thickness > 17 mm group, the POPF rate tended to be lower when a longer stapler was used, but this was not statistically significant (85.7% vs 82.6% vs 68.2%, *P* = 0.230). The OR between Group III and the others was 2.333 (95% of OR: 0.627–8.683, *P* = 0.206). The means of CR were 10.77 and 10.66 in the POPF (+), and POPF (−) groups, respectively (*P* = 0.833).

## 4 Discussion

POPF is the most serious complication in DP. In previous studies, BMI,^[6,7,10]^ thickness,^[7,8]^ and texture^[8,11]^ have been identified as risk factors for POPF after DP. Various efforts to reduce the POPF rate have been attempted, including perioperative treatment with octreotide analog, pasireotide,^[12]^ polyethylene glycolic acid mesh,^[13]^ topical absorbable fibrin sealant patch,^[14]^ pancreaticojejunostomy,^[15]^ falciform ligament patch,^[16]^ prolonged prefiring compression,^[17]^ and seromuscular patch.^[18]^ The method of pancreas stump closure is a major factor in preventing POPF.

Recently, stapling closure has become a popular method in DP due to its simplicity and safety compared with the conventional suture method. Some studies insist that the stapler is a safe method.^[4,19]^ Further studies have shown that closure with a stapler had a more favorable effect than conventional hand sewn closure.^[3,20]^ In a systematic review, the use of stapler closure in DP significantly reduces POPF rates compared with suture closure. The combination of stapler and suture was found to be superior over suture alone.^[2]^ However, in the DISPACT study,^[1]^ a multicenter, randomized, controlled trial in 21 European hospitals, the POPF rates of with stapler closure and hand-sewn closure were comparable. It concluded that stapler closure did not reduce the rate of POPF compared with hand-sewn closure for DP.

POPF could occur by various causes. When a short CL stapler is applied to a thick pancreas, POPF may develop due to rupture of the pancreas. To explain POPF developing in stapling closure, thickness should also be considered due to the compression effect. However, few studies have investigated the POPF rate according to the thickness and the stapler used. Some studies suggested that white stapler cartridge was optimal for DP.^[5,21]^ In other studies, the authors have described the superiority of gold or green cartridges in DP.^[22–24]^ However, these studies could not clarify the relationship between type of cartridges and POPF. Therefore, the purpose of this study was to find the optimal stapler cartridge according to the thickness in DP. Through this study, recommendation of customized stapler selection for each pancreas was expected.

In this study, the high POPF rate (overall POPF rate: 59.9%, clinically relevant POPF rate: 20.3%) compared with recent studies was due to our protocol for DP and data collection methods. A JP drain was placed in the pancreatic stump of every DP patient; drain amylase was checked at postoperative days 1, 3, and 5 routinely, and data were collected prospectively. Therefore, the Grade A POPF rate was higher than that seen at other institutes where drains are inserted selectively. CT scans were also checked routinely at postoperative day 5. Therefore, the chance of identification of fluid collection was high. This high detection rate of asymptomatic fluid collection contributed to the high POPF rate.

In this study, it was found out that the POPF rate increased as thickness increased. In subgroup analysis, gold cartridge (leg length: 3.8 mm, CL: 1.8 mm, Group II) was the optimal stapler cartridge in thin pancreases below 12 mm, rather than white which was shown as the optimal stapler in previous study.^[5,21]^ In thick pancreases, long CL cartridges Group III (green or back), had a tendency to show lower POPF rates, but this was statistically insignificant. In the overall group, stapler group did not show any difference between the POPF (+) and POPF (−) groups. An optimal stapler for general use in DP did not exist.

To explain the compression effect, CR defined as thickness/CL was introduced. It was expected that significant difference of CR wound be shown between POPF (+) and POPF (−) groups in subgroups according to the thickness, but this difference was not shown. In thickness below 12 mm, when a Group I stapler (short CL) was used, the pancreas was compressed more strongly compare to Group II staplers. On the contrary, when Group III staplers (long CL) were used, the pancreas was compressed weakly. This meant that POPF developed when the pancreas was compressed weakly (low CR) or strongly (high CR) in thin pancreases. Therefore, the mean of CR did not show any difference in the below 12 mm pancreas group. In thick pancreases, above 17 mm, there was no suitable stapler, and CR did not show any difference between the POPF (+) and POPF (−) groups.

These findings, with different results between thin and thick pancreases, were due to differences in risk factors according to the thickness. To compare risk factors according to thickness, thickness was split into 2 groups based on the median value, 15 mm. The effect of thickness was marginally significant in thin pancreases. In thick pancreases, however, the thickness effect was insignificant. In thin pancreases, thickness was important factor for POPF, so the stapler effect was significant. In thick pancreases, on the other hand, the effect of the length of cartridges on development of POPF was weak even with longer CLs. The POPF rate was consistently high in thick pancreases. As it is difficult to compress and divide the pancreas with even pressure with the current stapler, POPF might develop. Therefore, additional efforts including reinforcement suture are necessary in thick pancreases.

Texture of pancreas was regarded as a risk factor for POPF.^[8,21]^ In our results, hard pancreases had a tendency to develop POPF (*P* = 0.124). In contrast with pancreaticoduodenectomy in which soft pancreas is a risk factor,^[11]^ pancreaticojejunostomy was not a risk factor in DP. Effective compression was an important factor for DP. Soft pancreases were more easily compressed. Therefore, hard pancreases had a tendency to develop POPF. The most potent risk factor, however, was BMI as many previous studied have suggested.^[6,10,25]^ It might be difficult to perform a secure operative procedure, with increased risk of incomplete compression or minor duct injury in high BMI patients due to increased visceral fat and the small operative field.

There have been several studies about compression time. Prolonged compression time is effective for prevention of POPF in laparoscopic DP.^[23]^ In this study, the pancreas was compressed for over 3 min. This procedure was able to flatten the pancreas around the resection line. In our study, we could not measure the compression time. Usually, we compressed the pancreas for at least 20 to 30 s. Further prospective study is needed to determine the compression time.

Laparoscopic surgery is a popular method for DP, although the rate of open DP (58.5%) is high in this study. At our institute, open surgery is preferred for malignant disease. Malignant cases comprised 40.1% of all cases, including pancreatic ductal adenocarcinoma (33.6%); therefore, the rate of open DP was relatively high. The POPF rate in laparoscopic DP was higher than that in open DP; however, there was no statistical significance on multivariate analysis.

In conclusion, the POPF rate increased as pancreas thickness increased. Because the compression effect is a critical point in POPF development after DP with endoscopic stapler, it is important to choose the optimal stapler which will compress not too strongly and not to weakly. Gold cartridge was optimal stapler in thin pancreas. In thick pancreas, a longer staple height is recommended but further study is necessary to investigate how to reduce the POPF rate.
